# A quantitative assessment of the consequences of allowing dose heterogeneity in prostate radiation therapy planning

**DOI:** 10.1002/acm2.12424

**Published:** 2018-08-11

**Authors:** Lingyue Sun, Wendy Smith, Abhijit Ghose, Charles Kirkby

**Affiliations:** ^1^ Department of Physics and Astronomy University of Calgary Calgary AB Canada; ^2^ Department of Medical Physics Tom Baker Cancer Centre Calgary AB Canada; ^3^ Department of Oncology University of Calgary Calgary AB Canada; ^4^ Department of Radiation Oncology Jack Ady Cancer Centre Lethbridge AB Canada; ^5^ Department of Medical Physics Jack Ady Cancer Centre Lethbridge AB Canada

**Keywords:** OAR sparing, prostate VMAT planning, PTV upper dose constraint

## Abstract

Target dose uniformity has been historically an aim of volumetric modulated arc therapy (VMAT) planning. However, for some sites, this may not be strictly necessary and removing this constraint could theoretically improve organ‐at‐risk (OAR) sparing and tumor control probability (TCP). This study systematically investigates the consequences of PTV dose uniformity that results from the application or removal of an upper dose constraint (UDC) in the inverse planning process for prostate VMAT treatments. OAR sparing, target coverage, hotspots, and plan complexity were compared between prostate VMAT plans with and without the PTV UDC optimized using the progressive resolution optimizer (PRO, Varian Medical Systems, Palo Alto, CA). Removing the PTV UDC, the median D1cc reached 144.6% for the CTV and the PTV, and an average increase of 3.2% TCP was demonstrated, while CTV and PTV coverage evaluated by D99% was decreased by less than 0.6% with statistical significance. Moreover, systematic improvement in the rectum dose volume histograms was shown (a 5–10% decrease in the volume receiving 50% to 75% prescribed dose), resulting in an average decrease of 1.3% (*P* < 0.01) in the rectum normal tissue complication probability. Additional consequences included potentially increased dose to the urethra as evaluated by PTV D0.035cc (median: 153.4%), delivering 283 extra monitor units (MUs), and slightly higher degrees of modulation. In general, the results were consistent when a different optimizer (Photon Optimizer, Varian Medical Systems) was used. In conclusion, removing the PTV UDC is acceptable for localized prostate cases given the systematic improvement of rectal dose and TCP. It can be particularly useful for cases that do not meet the rectum dose constraints with the PTV UDC on. This comes with the foreseeable consequences of increased dose heterogeneity in the PTV and an increase in MUs and plan complexity. It also has a higher requirement for reproducing the position and size of the target and OARs during treatment. Finally, with the PTV UDC completely removed, in some cases the maximum doses within the PTV did approach levels that may be of concern for urethral toxicity and therefore in clinical implementation it may still be necessary to include a PTV UDC, but one based on limiting toxicity rather than enforcing dose homogeneity.

## 1 INTRODUCTION

The treatment planning goals of intensity modulated radiation therapy (IMRT) and volumetric modulated arc therapy (VMAT) are to give a specific and conformal dose to the prescribed target volume and limit the dose to the surrounding normal tissues and organs‐at‐risk (OARs) to acceptable levels. Besides, target dose uniformity has been a default objective during inverse planning for several reasons. Using a simple model of biological response, it can be shown that for a uniform distribution of clonogenic tumor cells, a uniform dose distribution is the optimal dose distribution for tumor control (assuming constant integral dose through the volume).^1^ It could also be argued that there are pressures in terms of historical consistency and clinical experience to maintain dose uniformity. Before 2010, it was recommended by the International Commission on Radiation Units and Measurements (ICRU) in Reports 50 and 62 that dose heterogeneity within the planning target volume (PTV) should be within the range of −5% to +7% of the prescribed dose.^2^, ^3^ In the more recent ICRU report 83, this constraint is not mandatory if normal tissue sparing is a greater concern.^4^ Clinical trial protocols for the treatment of prostate cancer commonly state maximum dose constraints for the PTV. A survey of recent clinical trial protocols for maximum dose constraints is shown in Table 1.

**Table 1:** A survey of maximum PTV constraints specified in clinical trial protocols involving prostate radiotherapy

Study	PTV definition	Volume of PTV	Max dose to PTV (percent of prescribed dose)
PROFIT^13^	Prostate ± SVs + 10 mm (7 mm posteriorly)	1 cc	105
RTOG 0126^14^	Prostate + proximal SVs + 5–10 mm	2%	107
Dutch Multicentre Dose Escalation Trial^15^, ^16^	Prostate ± SVs + 5 or 10 mm (0 mm posteriorly)	Mean dose to PTV	107
RTOG 0815^17^	Prostate + proximal SVs + 5–10 mm	0.03 cc	107
RTOG 0415^18^	Prostate + 4–10 mm	Maximum dose to PTV	107
CHHiP^19^	Prostate ± SVs + 10 mm (0 mm posteriorly)	1%	105
RTOG 0521^20^	Prostate + proximal SVs + 5–15 mm	3%	107

SVs, the seminal vesicles.

A recent letter from Craft et al.^5^ to the International Journal of Radiation Biology Oncology Physics has drawn the strict enforcement of dose homogeneity into question. In principle, when a target volume dose distribution is nonuniform, the clonogenic cells with the highest probability for survival should be those in the subvolumes receiving the lowest doses, and therefore the low‐dose tail of a heterogeneous distribution is more likely to dictate tumor control. This draws into question the reasons for limiting PTV dose on the high end. Certainly for target volumes containing relatively high quantities of nerves, blood vessels, or normal tissue stroma, restricting the maximum dose to the target volume is often necessary in order to control toxicity.^6^ Otherwise, enforcing restrictions on the high end of PTV dose uniformity may not be warranted. Disease sites where ablative doses have been proven effective such as prostate, liver, or sarcoma, may achieve equal or better tumor control if dose is escalated to subvolumes of the PTV.^7^ In radiation therapy modalities like brachytherapy, stereotactic body radiation therapy, and stereotactic radiosurgery, highly heterogeneous dose distributions are common within the target volume, which suggests that allowing heterogeneous dose distributions in an IMRT or VMAT context is not unreasonable. Craft et al.^5^ suggested that allowing for greater dose heterogeneity while ensuring the minimum prescribed dose to the target volume would, in general, better spare critical structures around the target volume in IMRT or VMAT plans. From a physics perspective, a combination of beam penumbra and scatter results in nonsharp dose profiles, and extending the field edges beyond the PTV border is typically done to ensure adequate coverage at the PTV periphery; however, allowing higher and nonuniform doses within the PTV core could achieve adequate coverage by putting the steepest part of the dose profile at PTV border, leading to lower dose to the normal tissue. From an optimization perspective, removing the PTV upper dose limit removes a constraint on the optimization problem, increasing the viable solution space, potentially allowing for improvements in the overall goals of the plan.

While Craft et al.^5^ presented an example pancreatic cancer plan, to our knowledge, the effect of enforcing PTV dose uniformity on OAR sparing has not been thoroughly and quantitatively studied yet. In this study, we looked specifically at the consequences of limiting the upper dose to the PTV for low‐ and intermediate‐risk prostate cancer VMAT treatment plans. We investigated this site because (a) ablative doses are generally acceptable for prostate radiation therapy,^8^, ^9^, ^10^, ^11^, ^12^ which suggests minimal risk of toxicity from overdosing the PTV exclusively (within reason), (b) it is a site suggested by Craft et al.,^5^ and (c) prostate treatments are extremely common and make up a major fraction of the clinical workload for many clinics. We conducted a treatment planning study with a cohort of 17 anonymized VMAT patients using our clinical protocol for prostate cancer patients and compared optimization with and without an upper dose constraint on the PTV, which would be thought to be the best case scenario in terms of OAR sparing. The resulting plans were compared in terms of OAR sparing, target coverage, hotspots, and a general assessment of plan complexity. The purpose of this study is to quantitatively evaluate the consequences of removing the PTV UDC.

## 2 MATERIALS AND METHODS

### 2.A Patient selection, contouring, and planning

As a treatment planning quality control and improvement exercise, data from 17 early‐to‐intermediate stage prostate patients who were successively treated with volumetric modulated arc therapy (VMAT) were selected and anonymized. All the patients were prescribed with 78 Gy in 39 fractions. The patients were imaged in supine position with a comfortably full bladder and empty rectum on a Philips Brilliance Big Bore (Philips Healthcare, Cleveland, OH) with a slice thickness of 3 mm using the same standard pelvic protocol.

The software DICOM+ (University of Michigan Radiation Oncology) was run to anonymize all the 17 image datasets. The clinical target volume (CTV) was defined as the prostate with (10 datasets) or without (seven datasets) proximal seminal vesicles. The planning target volume (PTV) was created with an expansion of 8 mm around the CTV, 6 mm posteriorly. The rectum was defined as inferiorly from the lowest level of the ischial tuberosities and superiorly to the rectosigmoid junction. The rectal planning organ‐at‐risk volume (PRV) was defined as the rectum with a 3 mm margin in all directions. The bladder was defined as inferiorly from its base and superiorly to the dome. No PRV margin was added to the bladder for the optimizations, as this is not common planning practice at our center. However, we did retrospectively consider dosimetric consequences to a bladder PRV defined as a uniform 3 mm expansion of the bladder. The overlap regions of PTV and rectal PRV as well as PTV and bladder were all contoured.

All cases were replanned with two full arcs in the treatment planning system (Eclipse version 13.6, Varian Medical System, Palo Alto, CA) using the progressive resolution optimizer (PRO). AcurosXB with a dose grid resolution of 2 mm was used for dose calculation in this study. Dose to medium was scored. Table 2 lists the dose objectives for the target volumes and OARs. Minor violations (±2.5% prescribed dose) of the dose objectives were allowed.

**Table 2:** Structures and dose objectives for prostate VMAT plans

Structure	Dose objectives
CTV	D99% > 100%;
PTV	D99% > 95%; D1cc < 105%
Bladder	V65Gy < 50%; V70Gy < 35%; V75Gy < 25%; V80Gy < 15%
Rectum	V50Gy < 50%; V60Gy < 35%; V65Gy < 25%; V70Gy < 20%; V75Gy < 15%
Femur	V50Gy < 5%

The optimization objectives were defined during the inverse planning process and they were parameters in the cost function. Optimization objectives were usually set to be tighter than the dose objectives especially for the structures that were harder to meet the dose objectives (example in Table 3). The optimization objectives were slightly adjusted on a per patient basis if necessary. All the VMAT plans for each individual patient had the same optimization objectives except for the presence/absence of the PTV upper dose constraint (UDC). The overlap regions of the PTV and rectal PRV or bladder also had an upper dose constraint to avoid hotspots in these regions. No adjustment to the optimization objectives was made during the process of optimization. Plan normalization was chosen such that 95% of the PTV received 100% of prescribed dose. All the plans were reviewed by a senior physicist and an experienced planner.

**Table 3:** Structures and optimization objectives for patient 1

Structure	Sample optimization objectives		
	Limit	Volume (%)	Dose (cGy)
PTV	Lower	100	7878
	Upper	0	7956
Bladder	Upper	50	6500
	Upper	35	7000
	Upper	25	7500
	Upper	15	8000
Rectal PRV	Upper	50	5000
	Upper	35	5500
	Upper	20	6000
	Upper	15	6500
Femur	Upper	5	4400
PTV and bladder/rectal PRV overlap region	Upper	0	7956

For every dataset, two VMAT plans were created:

- a plan with the PTV upper dose constraint using progressive resolution optimizer (PRO: with UDC) and,
- a plan without the PTV upper dose constraint using progressive resolution optimizer (PRO: without UDC).

To test the robustness of our results against the specific optimization algorithm, we repeated our methods and performed the same evaluations for plans with and without the PTV UDC optimized using a separate optimization algorithm, the photon optimizer (PO) in Eclipse (Eclipse version 13.6, Varian Medical System) for the 17 datasets.

### 2.B Plan modulation complexity score for VMAT plans (MCSv)

Removing a constraint in the optimization process has the potential to alter, on average, the complexity of the plans delivered by the linear accelerator. It is important to know if any improvements in OAR DVHs come with a consequence of an increased treatment plan complexity since more complex plans can be more difficult to deliver for the machine, take longer, or be more likely to fail patient‐specific QA. To this end, we calculated a modulation complexity score.

The modulation complexity score (MCS) was originally defined by McNiven et al.^13^ for evaluating IMRT plans. Later, Masi et al.^14^ applied it with modification to VMAT plans based on control points of an arc and it was found that MCS was closely correlated with VMAT dosimetric accuracy, which made it to be a candidate for scoring plan complexity.

The aperture area variability (AAV) is calculated as the area of the apertures of opposing leaves in the single control point (CP) normalized to the maximum area in the arc. The leaf sequence variability (LSV) takes into account the positional variations between adjacent MLC leaves in each bank relative to the maximum possible positional change in the CP. The modulation complexity score is based on the mean values of AAV_CP_ and LSV_CP_ weighted by the relative MUs delivered between two consecutive control points and then summed over all CP in the arc^16^, ^17^:

(1) $${\text{MCS}}_{\mathrm{arc}}=\sum_{i=1}^{I-1}\left[\frac{{\text{AAV}}_{{\mathrm{CP}}_{i}}+{\text{AAV}}_{{\mathrm{CP}}_{i+1}}}{2}\times \frac{{\text{LSV}}_{{\mathrm{CP}}_{i}}+{\text{LSV}}_{{\mathrm{CP}}_{i+1}}}{2}\times \left(\frac{{\text{MU}}_{{\mathrm{CP}}_{i+1,i}}}{{\mathrm{MU}}_{\mathrm{arc}}}\right)\right]$$

where ${\text{MU}}_{{\mathrm{CP}}_{i+1,i}}$ indicates the MU delivered between two successive control points. Plan modulation complexity score was performed by a MATLAB script (2016a, The MathWorks, Inc, Natick, Massachusetts, USA).

The ${\text{MCS}}_{\mathrm{arc}}$ has a value ranging from 0 to 1.^13^ When modulation increase*s,* ${\text{MCS}}_{\mathrm{arc}}$ decreases. ${\text{MCS}}_{\mathrm{arc}}=1$ indicates that the arc is delivered with a fixed rectangular aperture without the MLC leaves moving. MCSv is an average of ${\text{MCS}}_{\mathrm{arc}}$ for the two arcs.

Plans with and without the PTV upper dose constraint optimized using PRO were compared in terms of CTV and PTV coverage by D99%, CTV and PTV hotspots by D1cc, tumor control probability, homogeneity index (HI), which was defined as HI=(D2%−D98%)/D50%,^4^ the total number of monitor units (MUs), treatment time, plan modulation complexity scores, and dosimetric parameters for OARs as well as a rectal normal tissue complication probability. Statistical analysis was performed in MATLAB using two‐tail paired Student's *t*‐tests and a script was used to control the false discovery rate because of multiple testing by the Benjamini–Yekutieli method.^15^ *P* ≤ 5% is considered statistically significant.

## 3 RESULTS

**Figure 1:**
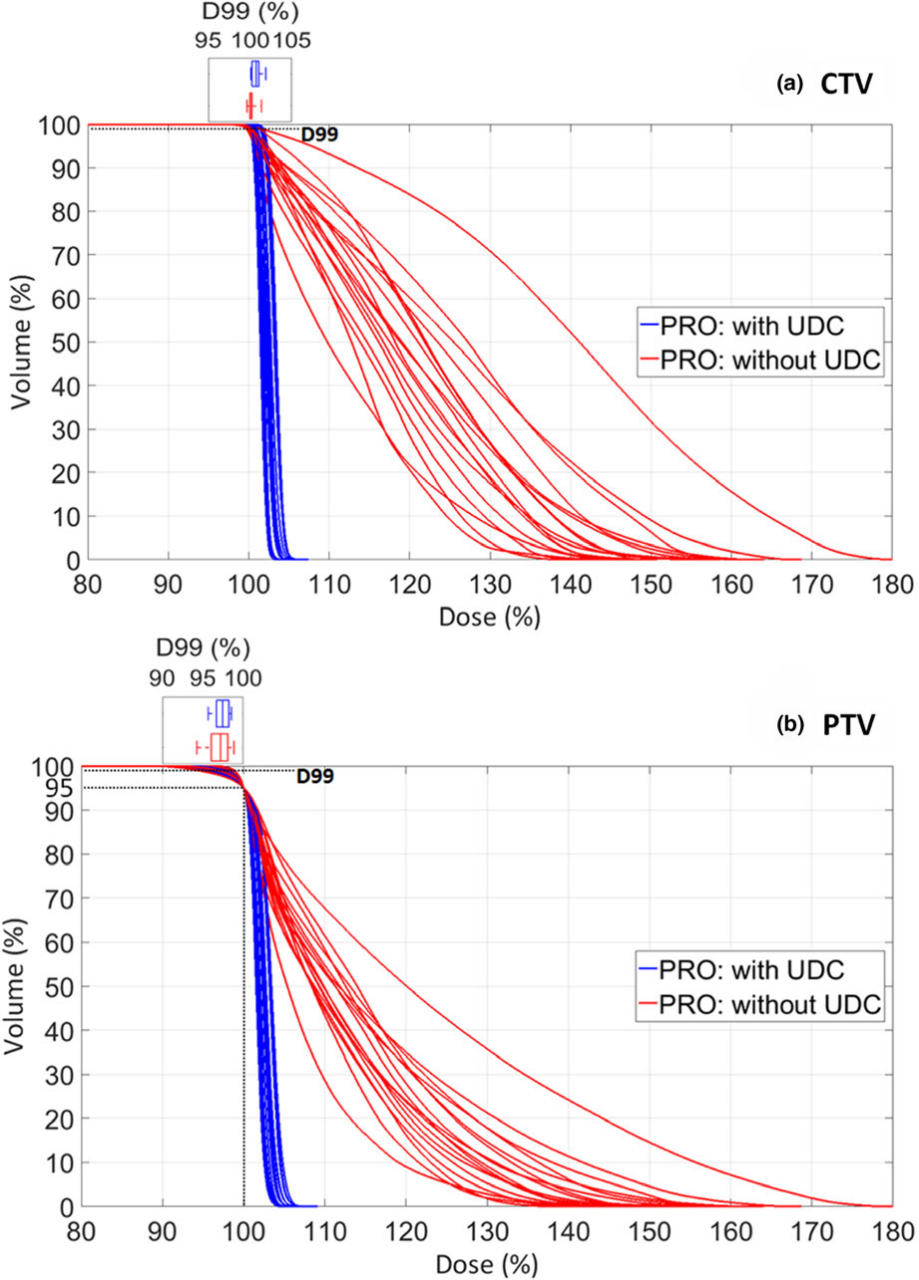
(a): CTV DVHs and boxplots for CTV D99%, (b): PTV DVHs and boxplots for PTV D99% from 17 datasets for plans with and without the PTV upper dose constraint (UDC) optimized using progressive resolution optimizer (PRO). Without the PTV UDC, the maximum dose received by CTV and PTV can be as high as 180% of prescribed dose and the CTV and PTV coverage evaluated by D99% was systematically lower with the normalization mode we used (100% of the prescribed dose to 95% of the PTV). However, the decrease in CTV and PTV D99% was too small to result in any clinically relevant impact.

Figure 1 is the CTV and PTV DVHs for plans with and without the PTV upper dose constraint (UDC) optimized using the PRO for the 17 datasets. Without the PTV UDC, the maximum dose of the CTV and PTV reached 135% to 180% of the prescribed dose, and nearly 50% to 75% of the PTV received a dose in excess of 105% of the prescribed dose.

First, we wanted to quantitate the consequences to the dose distribution within the target volume when the PTV UDC was removed. It is important to keep in mind that each plan was normalized such that 100% of the prescribed dose was delivered to 95% of the PTV [as can be seen from Fig. 1(b)]. Still, it was important to examine the consistency of the plans with respect to minimal coverage, which was quantified as the dose to 99% of the volume, D99%. Figure 1 shows the boxplots of CTV and PTV D99% of plans with and without the PTV UDC from the 17 datasets. The median of CTV D99% was 100.7% (range: 100.1–101.9%) for plans with UDC and 100.1% (range: 99.6–101.4%) for plans without UDC. A paired Student's *t*‐test showed that the differences were statistically significant (*P* = 0.01). Meanwhile, the median of PTV D99% was 97.4% (range: 95.7–98.5%) for plans with UDC and 97.2% (range: 94.2–98.8%) for plans without UDC. Again the differences are statistically significant (*P* = 0.02). While there were systematic decreases of both PTV and CTV D99% values, the dose objectives were still achieved or very close to being achieved, and the magnitude of the change was too small to result in any clinically relevant impact. Therefore, as one may expect, removing the PTV UDC had little effect on PTV or CTV coverage for the normalization method used (100% of the prescribed dose to 95% of the PTV).

**Figure 2:**
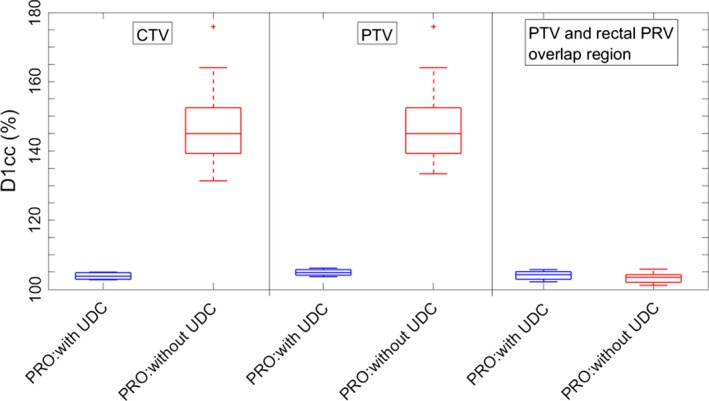
Boxplots of D1cc for CTV, PTV, and the overlap region of PTV and rectal PRV from 17 datasets for plans with and without the PTV upper dose constraint (UDC) optimized using progressive resolution optimizer (PRO). Without PTV UDC, the CTV and PTV D1cc increased dramatically and the median was still lower than 150% of prescribed dose. Moreover, removing PTV UDC significantly lowered D1cc for the overlap region of PTV and rectal PRV with a small amount. These results indicate that removing the UDC has no risk of overdosing the rectum.

To evaluate the hotspots in the CTV and PTV, the maximum dose received by 1 cc of the CTV and PTV, D1cc, was quantified. Figure 2 shows the boxplots of CTV and PTV D1cc of the two types of plans for the 17 datasets. The median CTV D1cc increased from 103.7% (range: 102.8–105.0%) for plans with the UDC to 144.6% (range: 131.3–164.0%) for plans without the UDC. Meanwhile, the median PTV D1cc increased from 104.9% (range: 103.7–106.2%) for plans with UDC to 144.6% (range: 133.4–164.0%) for plans without the UDC. On the other hand, removing the PTV UDC systematically lowered the D1cc for the overlap region of the PTV and rectal PRV by a small amount (*P* < 0.01). Median D1cc for the overlap region of the PTV and the rectal PRV was 104.2% (range: 102.2–105.8%) for plans with the PTV UDC and was 103.5% (range: 101.2–105.8%) for plans without the PTV UDC. By removing the PTV UDC, the CTV and PTV D1cc increased dramatically, but the hotspots stayed outside of the PTV and the rectal PRV overlap region with no risk of overdosing the rectum if an upper dose constraint was assigned for the overlap region during optimization process.

To quantitate the impact of the PTV UDC on target dose distribution, we translated the CTV DVHs into tumor control probability (TCP) for each of the plans of the 17 datasets using the Niemierko's EUD‐based model.^16^, ^17^, ^18^ A paired Student's *t*‐test was performed for plans with and without the UDC and the statistics are shown in Table 4. On average, plans without the UDC had a TCP 3.2% higher than plans with the PTV UDC with statistical significance as a result of the increased equivalent uniform dose (EUD).

**Table 4:** Statistics for EUD of CTV (Gy), TCP (%), EUD of rectum (Gy), and rectal NTCP (%)

Dataset^a^	EUD of CTV (Gy)		TCP (%)		EUD of rectum (Gy)		Rectal NTCP (%)	
	PRO: with UDC	PRO: without UDC	PRO: with UDC	PRO: without UDC	PRO: with UDC	PRO: without UDC	PRO: with UDC	PRO: without UDC
1	80.7	93.0	93.6	96.6	66.4	65.6	12.9	11.5
2	80.6	88.2	93.6	95.7	66.0	65.1	12.2	10.7
3	80.6	95.0	93.6	96.9	65.5	64.4	11.4	9.6
4	79.8	92.4	93.3	96.5	65.4	64.3	11.1	9.5
5	80.5	91.4	93.6	96.3	64.8	63.5	10.2	8.4
6	80.2	94.3	93.5	96.8	64.3	63.0	9.5	7.8
7	80.0	90.7	93.4	96.2	64.2	63.1	9.4	7.9
8	79.8	98.0	93.3	97.3	64.0	62.7	9.2	7.5
9	79.6	87.2	93.3	95.5	63.7	63.0	8.8	7.8
10	80.0	89.5	93.4	96.0	63.4	62.4	8.3	7.1
11	79.8	92.1	93.3	96.5	62.9	61.8	7.7	6.5
12	79.3	97.2	93.2	97.2	62.4	60.6	7.2	5.3
13	79.5	92.4	93.2	96.5	62.0	60.9	6.6	5.6
14	79.3	95.4	93.1	97.0	61.8	61.3	6.5	6.0
15	79.3	94.9	93.2	96.9	61.1	59.8	5.8	4.6
16	79.3	88.6	93.1	95.8	61.0	59.9	5.7	4.7
17	79.1	108.2	93.1	98.3	59.9	59.1	4.7	4.1
Average difference ± SD	13.6 ± 5.1		3.2 ± 0.8		−1.1 ± 0.3		−1.3 ± 0.4	
CI	(11.0, 16.2)		(2.8, 3.6)		(−1.2,−0.9)		(−1.5, −1.1)	
*P* value	<0.01		<0.01		<0.01		<0.01	

EUD, equivalent uniform dose; TCP, tumor control probability; NTCP, normal tissue complication probability; SD, standard deviation; CI, 95% confidence interval.

a Dataset number was ranked according to the rectal NTCP for plans with the PTV UDC. Datasets 1–6 were the datasets that did not meet the dose objectives for rectum with the PTV UDC applied. These, met or nearly met the objectives after the removal of the PTV UDC.

**Figure 3:**
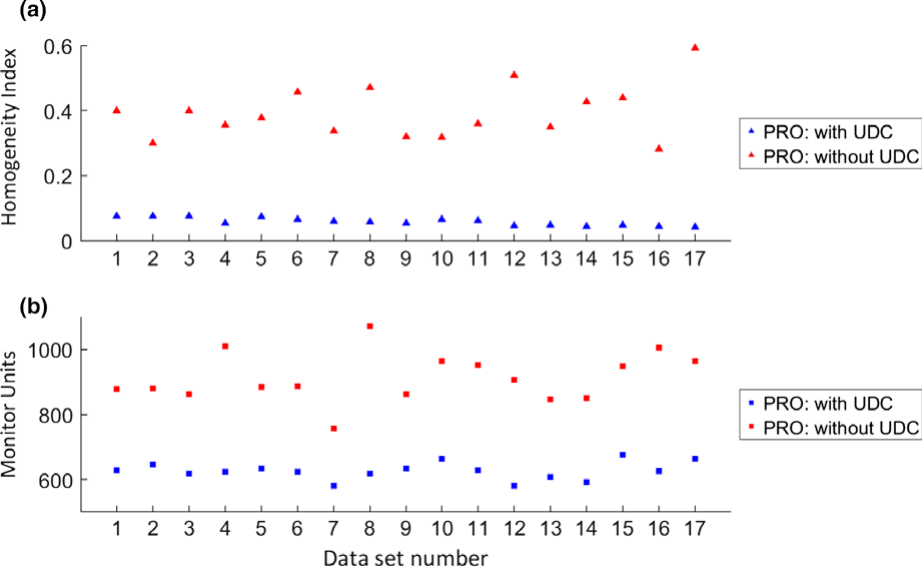
Scatter plots of the homogeneity index (HI) and monitor units (MUs) for the 17 datasets for plans with and without upper dose constraint (UDC) optimized using progressive resolution optimizer (PRO). On average, the HI increased 6.3 times (SD: 2.7 times) by removing the PTV UDC, and there was an increase of 283 MUs (SD: 70 MUs).

Figure 3(a) plots the homogeneity index (HI) of the two different plans for each patient dataset. Without the PTV UDC, the HI was on average 6.3 times larger than the HI of the corresponding plan with PTV UDC‐optimized PRO.

Secondly, plan complexity was assessed in terms of the number of monitor units (MUs), calculated delivery time, and plan modulation complexity scores (MCSv). Figure 3(b) shows the total number of MUs needed to deliver the plans with and without the PTV UDC for each dataset. On average, an extra 283 MUs (standard deviation: 70 MU) were needed to deliver the plan optimized without the PTV UDC. Interestingly, the time for delivering the two corresponding plans was similar, despite the increase in MUs.

Based on the model proposed by McNiven et al.^13^ and Masi et al.^14^ plan modulation complexity scores (MCSv) were calculated for each plan. A paired Student's *t*‐test was performed for plans with and without the UDC and the statistics from the 17 patient datasets are shown in Table 5. The plans without the PTV UDC were more complex with statistical significance (a smaller value of modulation complexity score meant a more complex plan). However, an average difference of 0.06 for MCSv is not likely to result in clinically relevant differences in terms of plan deliverability.^14^

**Table 5:** Statistics for the plan modulation complexity score (MCSv)

Difference of plan modulation complexity score	MCSv (PRO: without UDC) — MCSv (PRO: with UDC)
Number of datasets	17
Average ± SD	−0.06 ± 0.02
CI	(−0.07, −0.05)
*P* value	<0.01

SD, standard deviation; CI, 95% confidence interval.

**Figure 4:**
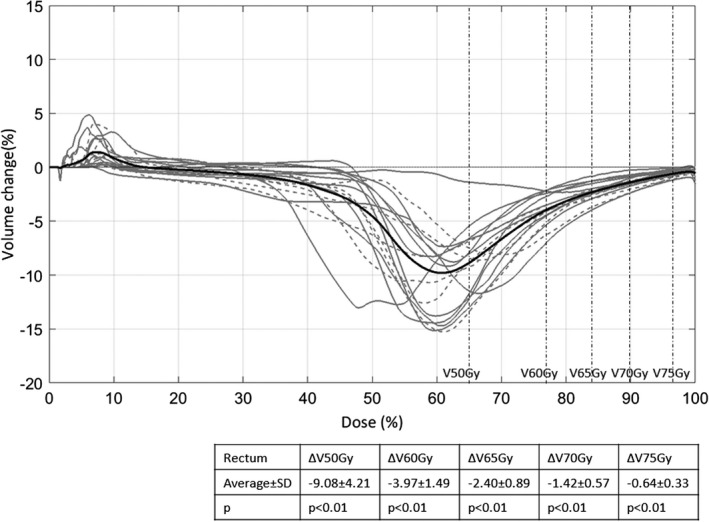
Difference in rectum DVHs for plans without and with the PTV upper dose constraint (UDC) for the 17 datasets. The gray curves were the rectum DVH difference for each of the datasets (the dashed ones were the datasets that did not meet the dose objectives for rectum with the PTV UDC on, but met or nearly met them after the removal of the PTV UDC) and the black curve was the average difference. Overall, removing the PTV UDC had a decreasing effect especially for the volume receiving a dose in the range of 50% to 75% prescribed dose. As shown in the table, there was a statistically significant decrease for the dosimetric parameters (V50Gy, V60Gy, V65Gy, V70Gy, and V75Gy) by removing the PTV UDC.

**Figure 5:**
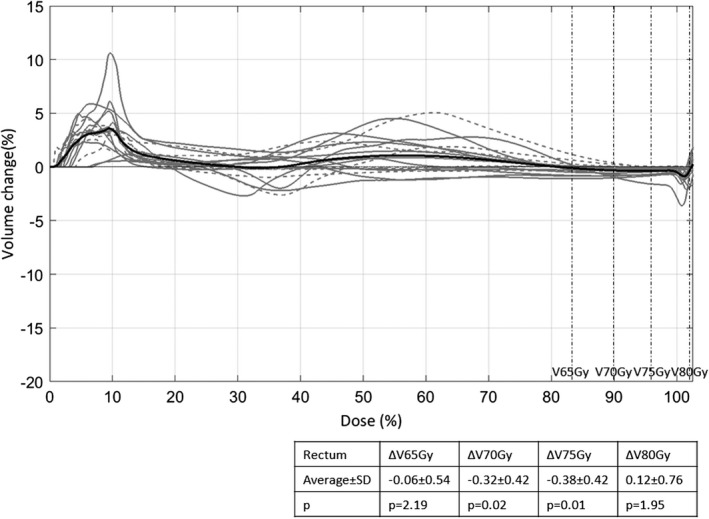
Difference in bladder DVHs for plans without and with the PTV upper dose constraint (UDC) for the 17 datasets. The gray curves were the bladder DVH difference for each of the datasets (the dashed ones were the datasets that did not meet the dose objectives for rectum with the PTV UDC on, but met or nearly met them after the removal of the PTV UDC) and the black curves was the average difference. As shown in the table, there was a statistically significant decrease in V70Gy and V75Gy after the removal of the PTV UDC. However, the decrease was too small to be clinically significant.

Thirdly, we evaluated the OAR sparing effect by comparing cumulative DVHs and dosimetric parameters. The DVH difference for plans without and with the PTV UDC (volume change vs dose) as well as the statistics for the dosimetric parameters is shown in Fig. 4 for rectum and in Fig. 5 for bladder for the 17 datasets. The volume change was calculated as the difference in percentage volume between the plan without the PTV UDC and the plan with the PTV UDC for each dose bin (0.1%). The closer the volume change was to zero, the less difference there was between the DVHs for the plans without and with the PTV UDC. A negative difference indicated the volume receiving that dose had decreased as a result of removing the PTV UDC. For rectum DVHs, on average, removing the PTV UDC had a decreasing effect, especially for the volume receiving a dose in the range of 50% to 75% of the prescribed dose (the black curve, which was an average of the 17 datasets). Dosimetric parameters of clinical interest for rectum were analyzed as shown in Fig. 4. All the dosimetric parameters, namely, V50Gy, V60Gy, V65Gy, V70Gy, and V75Gy decreased with statistical significance after removing the PTV UDC.

By performing a two‐tailed Pearson's linear correlation analysis between the rectum volume and the improvement of V50Gy, V60Gy, V65Gy, V70Gy, and V75Gy (in cc), we found there were moderate to strong correlations between them (*r* = 0.7 between ΔV50Gy and the rectum volume with *P* < 0.01; *r* = 0.8 between ΔV60Gy and the rectum volume with *P* < 0.01; *r* = 0.8 between ΔV65Gy and the rectum volume with *P* < 0.01; *r* = 0.8 between ΔV70Gy and the rectum volume with *P* < 0.01; *r* = 0.5 between ΔV75Gy and the rectum volume with *P* = 0.05). This indicates that a greater improvement of the dosimetric parameters in absolute volume (V60Gy, V65Gy, V70Gy, and V75Gy) would be expected for a patient who has a larger rectum volume.

In order to quantitate the impact of the upper dose constraint on rectal dose distributions, a common metric is needed. We chose to translate the rectal DVHs into a normal tissue complication probability (NTCP) as a metric representative of a clinical end point using Lyman's model.^19^, ^20^, ^21^ Table 4 also summarizes the rectal NTCP statistics from the 17 datasets. The average rectal NTCP for plans without the PTV UDC were 1.3% lower than plans with the PTV UDC, which was statistically significant.

For bladder in Fig. 5, removing the PTV UDC did not cause a great difference in DVHs although a statistically significant decrease was found for V70Gy and V75Gy. For these parameters, decreases of less than 0.4% are not likely to present any clinically relevant benefit.

The same analysis process was conducted for plans optimized using the PO algorithm. In general, the results were consistent between the optimization algorithms, although the PO algorithm tended to produce plans with smaller changes in MUs when the PTV UDC was removed (average change: 155 MUs, SD: 47 MUs) and there was a less decrease in the plan modulation complexity score (average change: 0.03, *P* < 0.01), compared to the PRO algorithm.

## 4 DISCUSSION

To assess the consequences of allowing increased dose heterogeneity in prostate radiation therapy, quantitative comparisons were made in this study between the plans with and without the PTV UDC optimized using the PRO algorithm. The results demonstrate that removing the PTV UDC, can offer systematic improvements in rectal DVHs and that these can translate into improvements in NTCP. As one might expect, maximum CTV and PTV dose and dose heterogeneity increase significantly. The most extreme PTV D1cc value was 176% of the prescribed dose, but in most cases this was less than 165%. The minimal coverage, as assessed by CTV and PTV D99%, remained acceptable. Hence, removing the PTV UDC ultimately increases dose heterogeneity through the PTV, but the increased levels of heterogeneity are not without clinical precedent. Comparable or greater levels of dose heterogeneity are typically seen in prostate brachytherapy.^22^, ^23^ Overall, the results are consistent between optimization algorithms.

The improvements in the rectum DVHs and dosimetric parameters without the PTV UDC (Fig. 4) are a direct result of the optimization process. Both PO and PRO are dose volume objective‐based optimizers. When the PTV UDC was removed, a larger portion of the cost function derived directly from the rectum constraints. Hence, if nothing else, the removal of the PTV UDC increases the relative weight of all other constraints. As suggested by Craft et al.,^5^ allowing higher doses in the PTV also offers the flexibility to generate coverage on the periphery of the PTV using the steepest part of the dose profile, ultimately lowering the dose outside the PTV compared with uniform target dose plans.

Importantly, there were six cases whose dosimetric objectives (i.e., one or more of V50Gy, V60Gy, V65Gy, V70Gy, and V75Gy) were not met for the plan with the PTV UDC applied, largely because of the overlap in volume between the PTV and rectal PRV. Without the PTV UDC applied, the optimizer was able to meet the specified constraints with one exception (dataset 2 resulted in V75Gy being reduced from 16.3% to 15.7% in attempting to meet a 15% volume objective when the PTV UDC was removed). These cases are datasets 1–6 in Table 4 and Fig. 3, and are depicted with dashed gray curves in Figs. 4 and 5.

In Fig. 4, there is one dataset in which the rectum DVH does not appear to improve as much as others, in particular for the volume that is receiving 50% to 75% of the prescribed dose. This patient had a lot of gas in the rectum. After assigning the HU value of the rectum a value of zero and rerunning the optimization process, we found there was a greater improvement (e.g., ΔV50Gy = −4.1%, ΔV60Gy = −2.8%). In a gas medium, the steepness of dose gradient is lessened, therefore removing the PTV UDC did not convey similar rectal DVH improvements to the other cases, but did when the HU values were set to zero.

For bladder, there was a very small variation when the PTV UDC was removed (Fig. 5). In general, for bladder, the optimization objectives were met even with the PTV UDC present. If the constraints were met, contributions to the cost function for the bladder in principle would equal zero and would not change when the PTV UDC was removed. Therefore, the optimization process tended to concentrate on improving the constraints yet to be met. The low to moderate dose volumes within the bladder DVH tended to increase slightly since they too were unconstrained. The number of volume elements receiving high dose remained the same or decreased slightly to maintain consistence with the planning constraints.

Improvements to the planned bladder DVH were modest, but this is also an organ that can move and change volume, and as such there is a question as the robustness of these results on delivery. We retrospectively created a bladder PRV (bladder + 3 mm) and assessed the dose to this structure. The dosimetric parameters for the bladder PRV structure were well within the dose objectives for bladder (V65Gy < 50%, V70Gy < 35%, V75Gy < 25%, V80Gy < 15%) in all 17 plans without PTV UDC. This suggests that minor changes in bladder position are unlikely to push the planning metrics beyond their constraints.

Recent studies have shown that changing the fractionation scheme to deliver higher doses per fraction can potentially increase bladder toxicity.^24^ Because removing the PTV UDC systematically increases the mean and maximum PTV dose, there may be concerns about increasing bladder toxicity even in light of modest improvements to the bladder DVH, although we would expect a number of factors to play a mitigating role. First, the existing hypofractionated studies deliver a relatively uniform dose to the whole PTV, presumably including any regions of overlap with the bladder. In our study, we limited the dose to the bladder and PTV overlap region to be less than 102% of the prescription (78 Gy in 39 fractions). It seems reasonable to expect any toxicity to correlate more strongly with planned dose to the overlap volume than with the PTV as a whole, when these values are different. Second, movement of portions of the bladder into the directly irradiated volume on delivery seems a reasonable factor for contributing to the reported increases in toxicity, but because removal of the PTV UDC tends not to increase dose on the PTV periphery, this factor would be mitigated. Third, removal of the PTV UCD may allow more flexibility to introduce and achieve tighter bladder dose constraints if those were to become a higher priority during the optimization process.

When the PTV UDC was removed, dose heterogeneity within the PTV was naturally expected to increase. In this study, we wanted to quantitate how much of an increase was reasonable to expect when the optimization algorithm was unrestricted on the high end and whether that increase pushed outside the realm of clinical experience across modalities. As shown in Fig. 3, HI increased 6.3 times on average, but always remained below a value of 0.6. The median D1cc of the CTV and PTV reached 144.6% using PRO (Fig. 2). Although these are certainly dramatic increases, they are not outside the realm of clinical experience. In prostate low‐dose rate brachytherapy, it is common for portions of the CTV to receive in excess of 200% of the prescribed dose. There are of course dose rate effects to consider, which may mitigate a direct comparison, but even if the HI results scaled up by a factor of 2 (i.e., D1cc values in the order of 200% of the prescribed dose) to obtain “brachytherapy equivalent” doses, the HI would still be less than that typically seen in brachytherapy prostate cancer treatments.

Removing the UDC increases the planned dose to regions of the PTV. With increased PTV dose comes a responsibility for increased vigilance in terms of setup and monitoring of patient position, as geographic miss‐type errors could potentially have more extreme consequences. It is important to note that the high doses we report with the PTV UDC removed are to small subvolumes of the PTV and the hotspots (D1cc) are in fact confined within the CTV (i.e., back from the outer periphery of the PTV). Because these subvolumes are relatively displaced from the bladder and rectum, the probability of a shifted bladder or rectum being exposed to these higher doses is relatively low. That said, the increased PTV dose may necessitate, for example, daily cone‐beam CT imaging which would include PTV alignment as well as assessments of the OAR positions and thresholds for treat/no treat decisions as a part of the setup protocol. This technique may be particularly well suited to hypo‐fractionated treatments where more detailed monitoring is already in place. It is also important that the UDC remains in place for the overlap region of the PTV and bladder as well as the overlap region of PTV and rectal PRV, and that the location of the hotspots relative to the OARs be scrutinized, as these will further help to reduce any differences between the planned and delivered dose distributions.

There is a statistically significant decrease in D1cc for the overlap region of the PTV and the rectal PRV (Fig. 2). The rectal PRV is a 3‐mm expansion from the rectum to account for the uncertainty in rectal position, relative to the target volume. Visual inspection of the spatial dose distribution in the plans with and without the PTV UDC shows that the overlap region is fully covered by 70 Gy isodose line and without the PTV UDC applied, the 70 Gy isodose does not “penetrate” as far into the rectal volume, which is why this manifests as a decrease in the rectal volume receiving 70 Gy. As a consequence, the rectum, which is perhaps the OAR of greatest concern for toxicities induced by prostate cancer radiation therapy, was not likely to receive a higher dose by removing the PTV UDC if an upper optimization objective was set for this overlap region.

As can be seen in Fig. 2 as well as Fig. 1, there is a case that has the most extreme gain in CTV and PTV D1cc (176.0%). This is from the patient who had the smallest overlap between the PTV and the rectal PRV. As a consequence, this case had the most freedom to escalate dose in the PTV (recall the PTV and rectal PRV overlap was still subject to an upper dose constraint), while working to meet the PRV objectives. Thus, for cases like this, removing the PTV UDC has the potential risk of giving the CTV and PTV a high dose that may not be acceptable.

Another potential consequence of increased dose to the PTV is increasing dose to the urethra. Because the urethra is not clearly visible in a CT scan, contouring and assigning constraints for the urethra during planning are not feasible using conventional approaches. A rough calculation can compare the urethral biological effective dose (BED) in EBRT to the dose in ^125^I implant low‐dose rate prostate brachytherapy according to Stock et al.^25^ For EBRT,

(2) BED=D∗(1+d/(α/β))

For ^125^I implant brachytherapy,

(3) $$\text{BED}=\left(R_{0}/\lambda \right)*\left[1+R_{0}/\left(\left(\mu +\lambda \right)\left(\alpha /\beta \right)\right)\right]$$

where $R_{0}=D*\lambda ,\lambda =0.693/T_{1/2},$ $T_{1/2}=60\;\text{days for}\;{}^{125}I\;\text{and}\;\mu =0.693/t_{1/2}$.^25^ From AAPM TG 137, for a prescription dose of 145 Gy for permanent ^125^I implants, it is recommended that final dose to 10% of the urethra should be <150% (218 Gy) for an acceptable toxicity rate.^22^ For fractionated EBRT, we assumed an alpha beta ratio of 5 Gy (some evidence suggests a range of 5–10 Gy for urethra late toxicity^26^) and tissue repair half‐life of 0.1 h as the worst case scenario. For conventional EBRT, this constraint translates to roughly 130 Gy for a 39 fraction EBRT treatment, or 165% of a 78 Gy prescribed dose. With the approximation that 10% of the urethra was 0.035 cc, we examined the PTV D0.035cc for the plans without the PTV UDC. It was found that in all but one of our results the PTV D0.035cc was 162.6% or less (median: 153.4%, range: 138.1–162.6%), and the worst case was 180.3% (this case was discussed previously).

The urethra toxicity rate is higher in brachytherapy than in EBRT.^27^ So when the optimization process is completely unconstrained on the upper end of the PTV (i.e., no PTV UDC), the maximum PTV doses have the potential to encroach on established urethral toxicity thresholds. Therefore, rather than completely eliminating the PTV UDC, users may wish instead to apply a PTV UDC based on urethral toxicity (e.g., PTV UDC in the range of 120–150% of prescription), which would still be expected to result in a moderate improvement for rectal dose while constraining the hotspots within the PTV to acceptable levels.

While CTV and PTV coverage was degraded slightly after the removal of the PTV UDC because of the normalization mode we used (100% of the prescribed dose to 95% of the PTV), it was still clinically acceptable (Fig. 1). Moreover, there was a 3.2% increase in TCP (Table 4). The increase in TCP results from an increase in an equivalent uniform dose across the CTV, since dose on the low end of the CTV DVH still must satisfy a lower dose constraint, but that on the high end increases as the UDC is removed. Conceptually, increasing maximum CTV dose to enhance TCP has been discussed in the literature. Goitein came up with the idea that delivering higher dose to a portion of the target volume could increase TCP.^7^ Balderson et al.^28^ investigated heterogeneous doses through a prostate PTV with modeling work that incorporated a bystander effect model derived from *in vitro* experiments,^29^ and concluded that a large amount of dose heterogeneity (a standard deviation of 10 Gy about a mean of 78 Gy) through a prostate target volume could be clinically acceptable, and a moderate amount may even be optimal in terms of EUD and TCP. Nielsen et al.^30^ claimed that inhomogeneous dose escalation in IMRT plans can potentially increase TCP by 10–15% for non‐small‐cell lung carcinoma patients without increasing lung toxicity compared with homogeneous plans.

As another consequence, the total number of MUs increased with statistical significance when the PTV UDC was removed as shown in Fig. 3. Interestingly, the calculated time needed for delivering the two kinds of plans did not change. In general, the dose rate increased to compensate for the increase in MUs. As shown in Table 5, the plan modulation complexity score decreased slightly after removing the PTV UDC, meaning an increase in plan complexity. Although this change was statistically significant, we would not expect the increased plan complexity to result in an increase in dosimetric errors between the delivered dose and the treatment planning system calculated dose for patient‐specific QA.^13^, ^14^ Plans optimized using the PO algorithm showed an even smaller decrease in plan complexity. This is probably due to the internal differences between the two optimizers. For PRO, every structure is represented by its own point cloud and dose is calculated for every dose point of each structure with a different resolution, while PO uses a consistent structure model where each structure location, DVH calculation and dose sampling are defined spatially by using one single matrix over the image.^31^, ^32^ In general the PO algorithm resulted in more complex plans to begin with but less complexity change. Overall, it seems that the specific optimization algorithm had little effect on the results.

## 5 CONCLUSION

In this study, we made quantitative comparisons between prostate VMAT plans with and without the PTV UDC optimized using two separate optimization algorithms, PRO and PO. With the PTV UDC removed, an average increase of 3.2% (*P* < 0.01) in tumor control probability was shown as a result of increased equivalent uniform dose. Removing the PTV UDC also systematically lowered the dose to the rectum as indicated by the general DVH differences and improvements in specific DVH points of interest (average improvement for V50Gy, V60Gy, V65Gy, V70Gy, and V75Gy was 9.1%, 4.0%, 2.4%, 1.4%, and 0.6%, respectively, *P* < 0.01). This led to an average decrease of 1.3% (*P* < 0.01) in the rectal normal tissue complication probability. There was no risk of overdosing the rectum. In fact, D1cc for the overlap region of the PTV and rectal PRV was systematically lower without the PTV UDC (note that an upper dose constraint was still applied to the overlap volume of the PTV and rectal PRV when it was removed for the PTV). On the other hand, these benefits came with costs. D99% for CTV and PTV was reduced, albeit by less than 0.6% (*P* = 0.01 and 0.02, respectively). We observed a 6.3 times larger heterogeneity index in the PTV, delivered an extra 283 MUs on average, and observed an average increase of 0.06 (*P* < 0.01) in plan modulation complexity score. PTV D0.035cc was examined to evaluate the possible maximum dose to the urethra after the PTV UDC was removed. In 16 of 17 patients, the PTV D0.035cc was less than 165% of the 78Gy prescription dose, the equivalent maximum tolerance dose of the urethra in prostate permanent seed implant as recommended by AAPM TG 137. To mitigate concerns about urethral toxicity, in practical implementation, users could still apply a PTV UDC, but one based on urethral toxicity limited rather than enforced dose homogeneity (e.g., a PTV UDC in the range of 120–150% of the prescription dose), which would still increase the freedom in the optimization process. The results were generally consistent between the two optimizers. Therefore, based on the evidence in this work, we conclude that removing the PTV UDC or basing it on urethral toxicity rather than enforcing dose heterogeneity offers moderate, but significant, planning advantages and it could be particularly useful for patients who are not meeting the rectum dose objectives with the PTV UDC applied.

## CONFLICT OF INTEREST

The authors declare that they have no conflict of interest.
